# Significant photocatalytic decomposition of malachite green dye in aqueous solutions utilizing facilely synthesized barium titanate nanoparticles

**DOI:** 10.1186/s11671-023-03873-x

**Published:** 2023-07-28

**Authors:** Asma S. Al-Wasidi, Ehab A. Abdelrahman

**Affiliations:** 1 Department of Chemistry, College of Science, Princess Nourah Bint Abdulrahman University, Riyadh, 11671 Saudi Arabia; 2 Department of Chemistry, College of Science, Imam Mohammad Ibn Saud Islamic University (IMSIU), Riyadh, 11623 Saudi Arabia; 3 Chemistry Department, Faculty of Science, Benha University, Benha, 13518 Egypt

**Keywords:** Photocatalytic decomposition, Barium titanate, Malachite green dye, Nanoparticles

## Abstract

The release of malachite green dye into water sources has detrimental effects on the liver, kidneys, and respiratory system. Additionally, this dye can impede photosynthesis and disrupt the growth and development of plants. As a result, in this study, barium titanate nanoparticles (BaTiO_3_) were facilely synthesized using the Pechini sol–gel method at 600 °C (abbreviated as EA600) and 800 °C (abbreviated as EA800) for the efficient removal of malachite green dye from aqueous media. The Pechini sol–gel method plays a crucial role in the production of barium titanate nanoparticles due to its simplicity and ability to precisely control the crystallite size. The synthesized barium titanate nanoparticles were characterized by several instruments, such as X-ray diffraction (XRD), high-resolution transmission electron microscopy (HR-TEM), Fourier transform infrared spectroscopy, and a diffuse reflectance spectrophotometer. The XRD analysis confirmed that the mean crystallite size of the EA600 and EA800 samples is 14.83 and 22.27 nm, respectively. Furthermore, the HR-TEM images confirmed that the EA600 and EA800 samples exhibit irregular and polyhedral structures, with mean diameters of 45.19 and 72.83 nm, respectively. Additionally, the synthesized barium titanate nanoparticles were utilized as catalysts for the effective photocatalytic decomposition of malachite green dye in aqueous media. About 99.27 and 93.94% of 100 mL of 25 mg/L malachite green dye solution were decomposed using 0.05 g of the EA600 and EA800 nanoparticles within 80 min, respectively. The effectiveness of synthesized BaTiO_3_ nanoparticles as catalysts stems from their unique characteristics, including small crystallite sizes, a low rate of hole/electron recombination owing to ferroelectric properties, high chemical stability, and the ability to be regenerated and reused multiple times without any loss in efficiency.

## Introduction

Presently, the contamination of water stands as one of the most urgent challenges confronting society, as it has a negative impact on the ecological balance. Due to the accelerated development of the industrial and agricultural sectors, our water bodies are severely polluted by the release of organic pollutants such as pesticides, dyes, insecticides, and other toxic organic compounds [1–5]. Malachite green dye poses potential dangers to humans, plants, and animals due to its toxic properties. In humans, direct exposure to malachite green dye can lead to cancer and have adverse effects on the liver, kidneys, skin, and respiratory system. The malachite green dye can inhibit photosynthesis and disrupt plant growth and development. In aquatic environments, it can be toxic to fish, amphibians, and other aquatic organisms, causing damage to their organs and impairing their overall health. Animals that consume contaminated water or come into direct contact with malachite green may experience adverse effects such as tissue damage and reproductive issues [6–8]. In this perspective, water treatment is required for the survival of a healthy living system [9–14]. Numerous techniques, including biological remediation, membrane filtration, adsorption, and catalytic oxidation, are utilized to remove organic contaminants from aqueous solutions. Among these methods, semiconductor photocatalysis is one of the most effective methods for decontaminating polluted water [15–21]. The photocatalytic activity of metal oxides such as ZnO, TiO_2_, ZrO_2_, ZnS, CuO, MgFeCrO_4_, Mg_0.5_Zn_0.5_FeMnO_4_, CoMnCrO_4_, Ni–Cu–Zn ferrite, and Ni_0.25_Zn_0.75_Fe_2_O_4_ is well known [22–31]. Zinatloo-Ajabshir et al. prepared a lot of photocatalysts such as Nd_2_Sn_2_O_7_, Dy_2_Sn_2_O_7_, ZrO_2_, CoFe_2_O_4_/SiO_2_/Dy_2_Ce_2_O_7_ composite, ZnCo_2_O_4_, and Nd_2_O_3_ for the degradation of several pollutants such as erythrosine, crystal violet, acid violet 7, eriochrome black T, methyl violet, rhodamine B, acid red 14, methylene blue, and eosin Y dyes [32–37]. It has been reported that spinel ferrites such as CuFe_2_O_4_ and ZnFe_2_O_4_ and pervoskite compounds such as BiFeO_3_, SrFeO_3_, and LaFeO_3_ have photocatalytic properties [38–40]. TiO_2_ is widely used as a photocatalyst due to its high catalytic efficiency, low cost, and high chemical stability [41]. In photocatalytic reactions, semiconductors can be excited by light energy exceeding their band gap, resulting in the formation of electron/hole pairs [42–44]. These photoinduced charge carriers can participate in redox reactions with contaminants [45, 46]. To achieve high photocatalytic performance, the recombination of holes and electrons must be avoided. Most semiconductors have a large band gap and a high recombination rate, which reduces the efficiency of the photocatalytic reaction. Heterojunction coupling and doping with metal ions are necessary to resolve the aforementioned issues [47, 48]. In contrast to TiO_2_, titanate-based compounds such as BaTiO_3_, SrTiO_3_, and CaTiO_3_ have intrinsic chemical reactivity. A few of them exhibit chemical stability and excellent photocatalytic activity. Due to their piezoelectric, pyroelectric, and ferroelectric properties, pervoskite compounds have garnered considerable interest [49]. Due to its ferroelectric property, BaTiO_3_ is among the most effective photocatalyst candidates. BaTiO_3_ has a direct band gap of 3.20 eV and is employed in electro-optical devices, thermistors, and transducers [50]. The band-bending property of ferroelectric material reduces charge carrier recombination, thereby improving its photocatalytic performance [51]. Rhodium-doped BaTiO_3_ was synthesized by Maeda and found application in hydrogen evolution [52]. Liu et al. reported that BaTiO_3_ in the form of a flower was used to degrade methyl orange dye [53]. Ren et al. synthesized electrospun fiber of BaTiO_3_/ZnO heterostructures using a combination of hydrothermal and electrospinning processes, and photocatalytic activities were investigated by the degradation of methyl orange dye [54]. The Pechini sol–gel process is a versatile and commonly used technique for the synthesis of a diverse range of nanomaterials such as Mn_0.5_Zn_0.5_Fe_2_O_4_/Fe_2_O_3_, MgMn_2_O_4_/Mn_2_O_3_, Fe_0.5_Mn_0.5_Co_2_O_4_/Fe_2_O_3_, MgMn_2_O_4_/Mn_2_O_3_/Mg_6_MnO_8_, and MgFe_2_O_4_ [46, 55, 56]. In this work, barium titanate (BaTiO_3_) nanoparticles with low crystallite sizes and different morphologies were facilely synthesized using the Pechini sol–gel technique. In addition, the synthesized nanoparticles were operated as photocatalysts for the efficient decomposition of malachite green dye in the presence of ultraviolet (UV) light. Besides, the effects of pH, irradiation time, quantity of catalyst, primary concentration of malachite green dye, scavengers, regeneration, and reusability were also investigated. The first part of innovation in our research comes from our ability to facilely synthesize BaTiO_3_ nanoparticles with very small crystal sizes using the Pechini sol–gel method. Furthermore, the second part of innovation in our research comes through the use of the synthesized nanoparticles as photocatalysts for the effective decomposition of malachite green dye, which is considered one of the most dangerous pollutants to the environment and humans. The synthesized BaTiO_3_ nanoparticles work as effective photocatalysts because they are distinguished by their small crystal sizes, low hole/electron recombination rate, and high chemical stability, as well as the ease of regenerating and reusing them many times without losing their efficiency.

## Experimental

### Chemicals

Titanium isopropoxide (C_12_H_28_O_4_Ti), ascorbic acid (C_6_H_8_O_6_), barium nitrate (Ba(NO_3_)_2_), ethylenediaminetetraacetic acid disodium salt dihydrate (C_10_H_18_N_2_Na_2_O_10_), tartaric acid (C_4_H_6_O_6_), isopropyl alcohol (C_3_H_8_O), sodium hydroxide (NaOH), ethanol (C_2_H_5_OH), malachite green dye (C_23_H_25_ClN_2_), hydrochloric acid (HCl), and ethylene glycol (C_2_H_6_O_2_) were of high analytical quality (Analytical grade), obtained from Sigma-Aldrich company, and used without purifying.

### Synthesis of barium titanate nanoparticles via the Pechini sol–gel process

The titanium tartrate/ethylene glycol network was freshly prepared by dissolving 3.50 g of titanium isopropoxide in 50 mL of ethanol. Subsequently, a tartaric acid solution was prepared by dissolving 3.69 g of tartaric acid in 50 mL of distilled water. The solution was then added to the titanium isopropoxide solution with continuous stirring for a duration of 20 min. Then, 6 mL of ethylene glycol was added, and the mixture was subjected to continuous stirring for 20 min. The barium tartrate/ethylene glycol network was synthesized by dissolving 3.22 g of barium nitrate in 50 mL of distilled water. Subsequently, a tartaric acid solution was prepared by dissolving 3.69 g of tartaric acid in 50 mL of distilled water. The solution was then added to the barium nitrate solution with a continuous stirring for a duration of 20 min. Then, 6 mL of ethylene glycol was added, and the mixture was subjected to continuous stirring for 20 min. After that, the titanium tartrate/ethylene glycol solution was added to the barium tartrate/ethylene glycol solution; then, the mixture was contentiously stirred at 130 °C until all the solvent had evaporated. The remaining powder was calcinated in a furnace at 600 and 800 °C for the decomposition of organic parts. The samples, which were synthesized at 600 and 800 °C, were abbreviated as EA600 and EA800, respectively.

### Characterization

X-ray powder diffraction (XRD) analysis of the EA600 and EA800 samples was conducted with a Panalytical Xpert Pro diffractometer equipped with K_α_Cu radiation (*λ* = 0.15 nm) at 30 mA and 45 kV. High-resolution transmission electron microscopy (HR-TEM) images of the EA600 and EA800 samples were taken using Talos F200iS microscopy. The FT-IR analysis of the EA600 and EA800 products was conducted with a Nicolet spectrometer. A Jasco V-750 diffuse reflectance spectrophotometer was used to determine the optical energy gap of the EA600 and EA800 products. The thermogravimetric analysis of the EA600 and EA800 products was performed using a LABSYS evo–SETARAM instruments. The HACH DR 5000 UV/Vis spectrophotometer was used to determine the concentration of the malachite green dye at its maximum wavelength of 622 nm.

### Photocatalytic decomposition of malachite green dye using the synthesized barium titanate nanoparticles

To assess the photocatalytic activity of barium titanate nanoparticles, malachite green dye was decomposed in the presence of three identical ultraviolet lamps (wavelength = 240 nm). To attain adsorption/desorption equilibrium, 0.05 g of barium titanate was added to 100 mL of a 25 mg/L malachite green dye aqueous solution, and the suspension was continuously stirred for a duration of 2 h in a dark environment. Additionally, the obtained mixture was then irradiated with ultraviolet light for a certain time. After that, the barium titanate nanoparticles were separated by centrifugation, and the concentration of malachite green dye in the filtrate was determined using a UV–Vis spectrophotometer. Moreover, investigations were conducted to examine the impact of various factors, including irradiation time (10–100 min), solution pH (3–9), amount of barium titanate nanoparticles (0.0125–0.20 g), and primary concentration of malachite green dye (15–35 mg/L). Additionally, the percentage of the photocatalytic activity (% *Z*) of EA600 and EA800 samples towards malachite green dye is estimated by Eq. (1) [14, 15].

1 $$\% Z = \frac{{C_{{\text{d}}} - C_{{\text{e}}} }}{{C_{{\text{d}}} }} \times 100$$

where *C*_d_ (mg/L) represents the resultant concentration of malachite green dye after being adsorbed in a dark environment. *C*_e_ (mg/L) denotes the concentration of malachite green dye after being exposed to ultraviolet light.

## Results and discussion

### Characterization of barium titanate nanoparticles

**Scheme 1.**
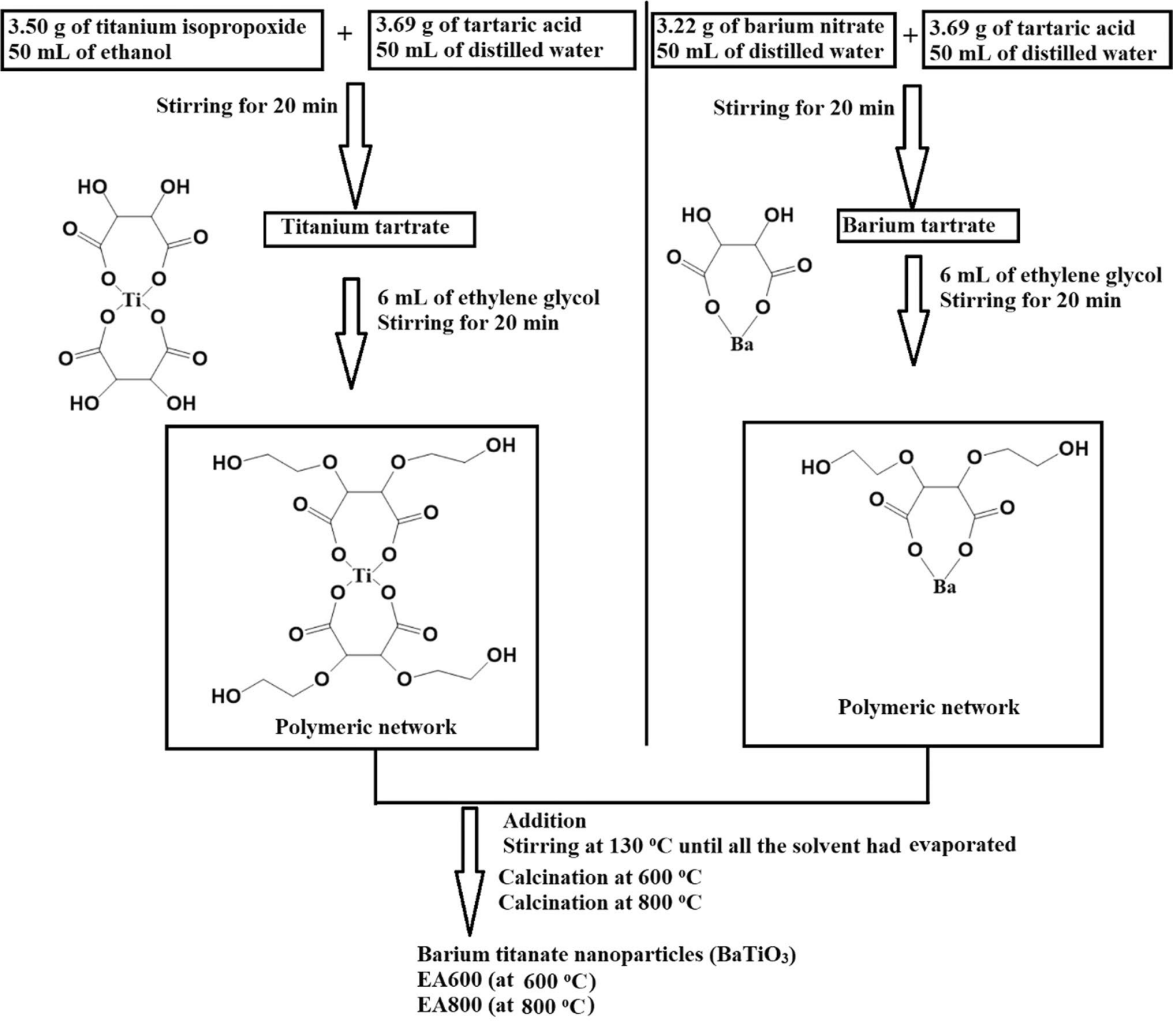
The synthesis of BaTiO_3_ nanoparticles using the Pechini sol–gel method

The Pechini sol–gel technique was employed for the synthesis of barium titanate nanoparticles. In this regard, titanium tartrate and barium tartrate were formed via the reaction of tartaric acid with titanium isopropoxide and barium nitrate, respectively. Ethylene glycol plays a vital role in the Pechini sol–gel method by serving as a polymerization control agent and participating in the gel formation process, as shown in Scheme 1. Ethylene glycol participates in the gelation process by reacting with tartaric acid. These reactions result in the formation of a three-dimensional gel network. The gel acts as a template for the subsequent formation of the final photocatalysts via calcination at 600 and 800 °C, as shown in Scheme 1 [17].

**Fig. 1:**
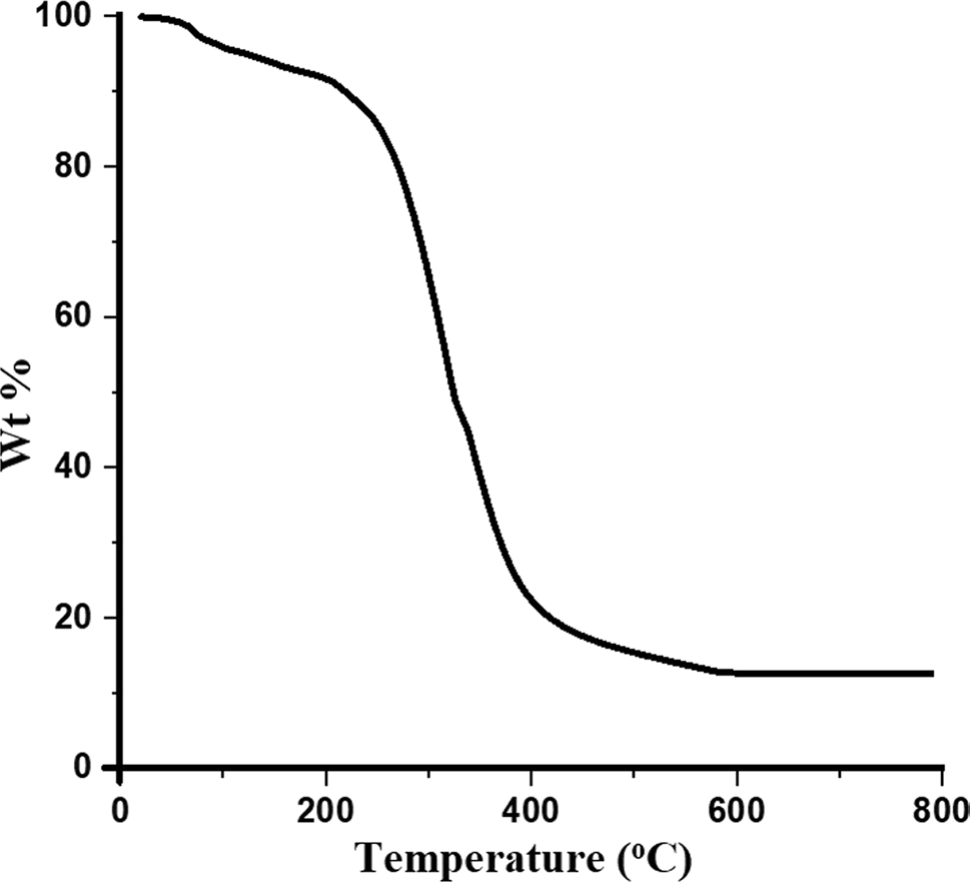
The thermogravimetric analysis of the resultant powder before calcination

The organic components need to be completely decomposed and removed to obtain the desired pure BaTiO_3_ nanoparticles. The chosen temperatures of 600 °C and 800 °C are likely suitable for the complete decomposition and removal of the organic species, as shown from the thermogravimetric analysis of resultant powder before calcination (Fig. 1).

**Fig. 2:**
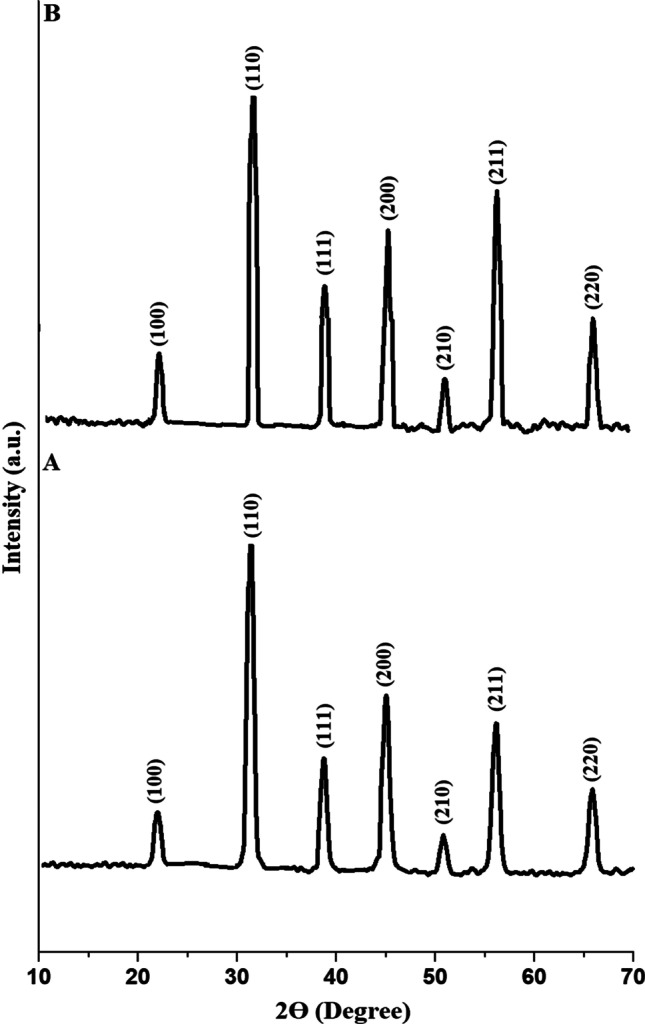
The XRD patterns of the EA600 (A) and EA800 (B) samples

The fingerprint property of XRD refers to the distinct pattern of diffraction peaks generated by a material, which serves as a unique signature for its crystal structure. These diffraction peaks correspond to the arrangement of atoms within the crystal lattice, providing valuable information about the material's composition and crystalline phases. Figure 2A and B displays the XRD patterns of the EA600 and EA800 samples, respectively. The XRD patterns shown in Fig. 1 exhibit prominent characteristic sharp peaks of barium titanate only, suggesting that the barium titanate nanoparticles possess a pure crystalline structure, as clarified from JCPDS No. 01-089-1428 [57]. The obtained diffraction peaks at 2*θ* = 66.24°, 56.50°, 51.27°, 45.39°, 39.15°, 31.89°, and 22.44° were ascribed to the (220), (211), (210), (200), (111), (110), and (100) miller planes of BaTiO_3_ nanoparticles, respectively, as clarified from JCPDS No. 01-089-1428 [57]. The mean crystallite size, which was determined using the Scherrer equation [58, 59], of the EA600 and EA800 samples is 14.83 and 22.27 nm, respectively. Hence, the results confirmed that as the calcination temperature increases, the mean crystallite size increases. The higher temperatures provide more thermal energy, which promotes the growth of crystals by enhancing diffusion and facilitating the rearrangement of atoms in the material, resulting in larger crystal sizes. Thus, this method plays an important role in controlling the crystallite size of barium titanate nanoparticles due to its unique characteristics and process parameters.

**Fig. 3:**
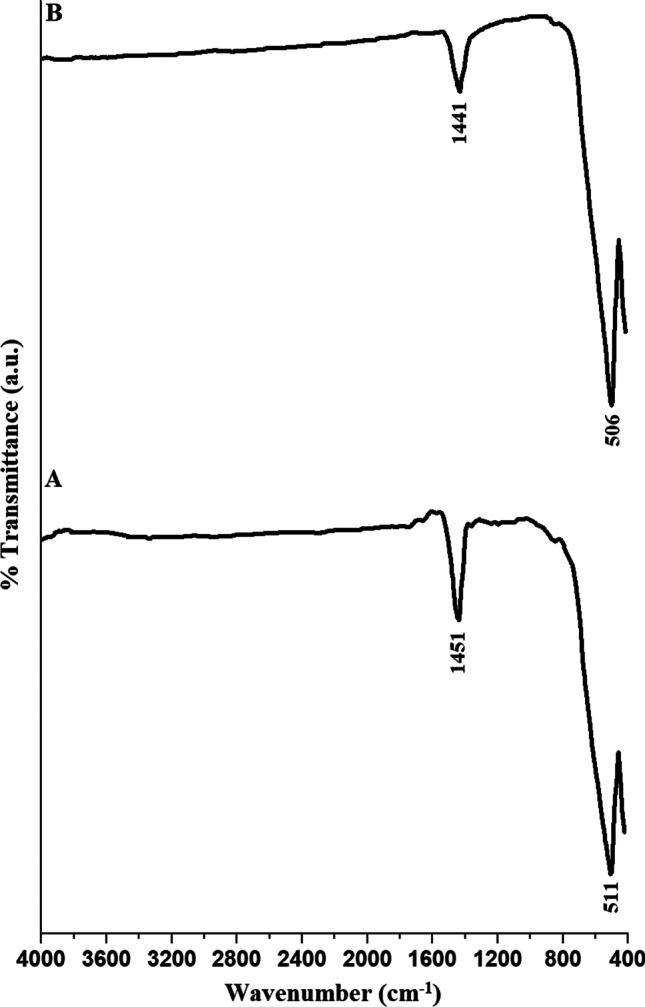
The FT-IR spectra of the EA600 (A) and EA800 (B) samples

Figure 3A and B displays the FT-IR spectra of the EA600 and EA800 samples, respectively. The bands, which were observed in the EA600 and EA800 samples at 511 and 506 cm^−1^, are due to the stretching vibrations of Ti–O, respectively [57, 60]. The bands, which were observed in the EA600 and EA800 samples at 1451 and 1441 cm^−1^, are due to the stretching vibrations of Ba-Ti–O, respectively [57, 60].

**Fig. 4:**
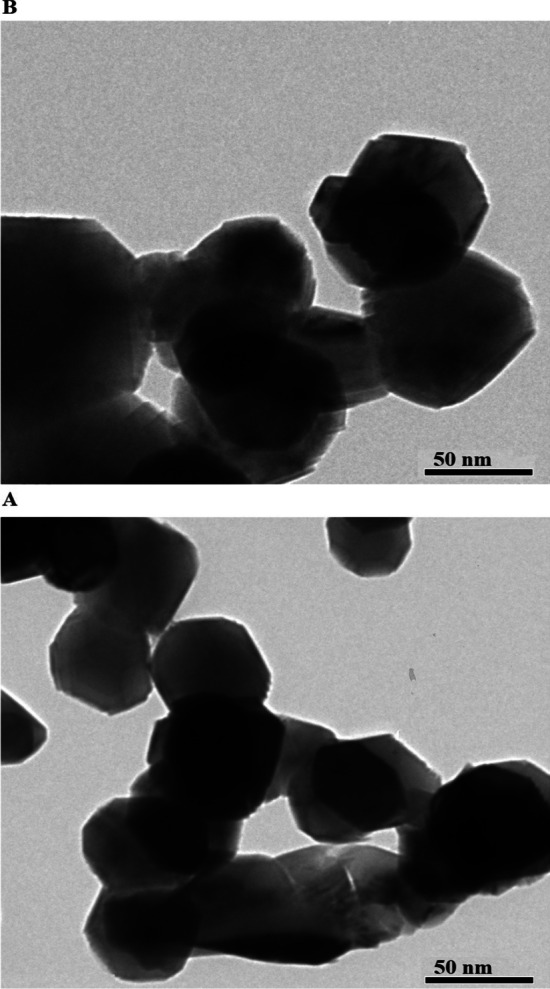
The HR-TEM images of the EA600 (**A**) and EA800 (**B**) samples

Figure 4A and B displays the HR-TEM images of the EA600 and EA800 samples, respectively. The findings verified that the EA600 and EA800 samples possess irregular and polyhedral structures, exhibiting average diameters of 45.19 and 72.83 nm, respectively. The agglomeration or clustering of particles can contribute to the inconsistency of crystallite size measurements obtained from X-ray diffraction (XRD) and transmission electron microscopy (TEM). Equation (2) is employed to determine the optical energy gap (*E*_g_) of the EA600 and EA800 samples by analyzing their diffuse reflectance spectra [45, 46].

2 $$\left( {F\left( R \right)h\upsilon } \right)^{N} = K_{{\text{E}}} \left( {h\upsilon - E_{{\text{g}}} } \right)$$

**Fig. 5:**
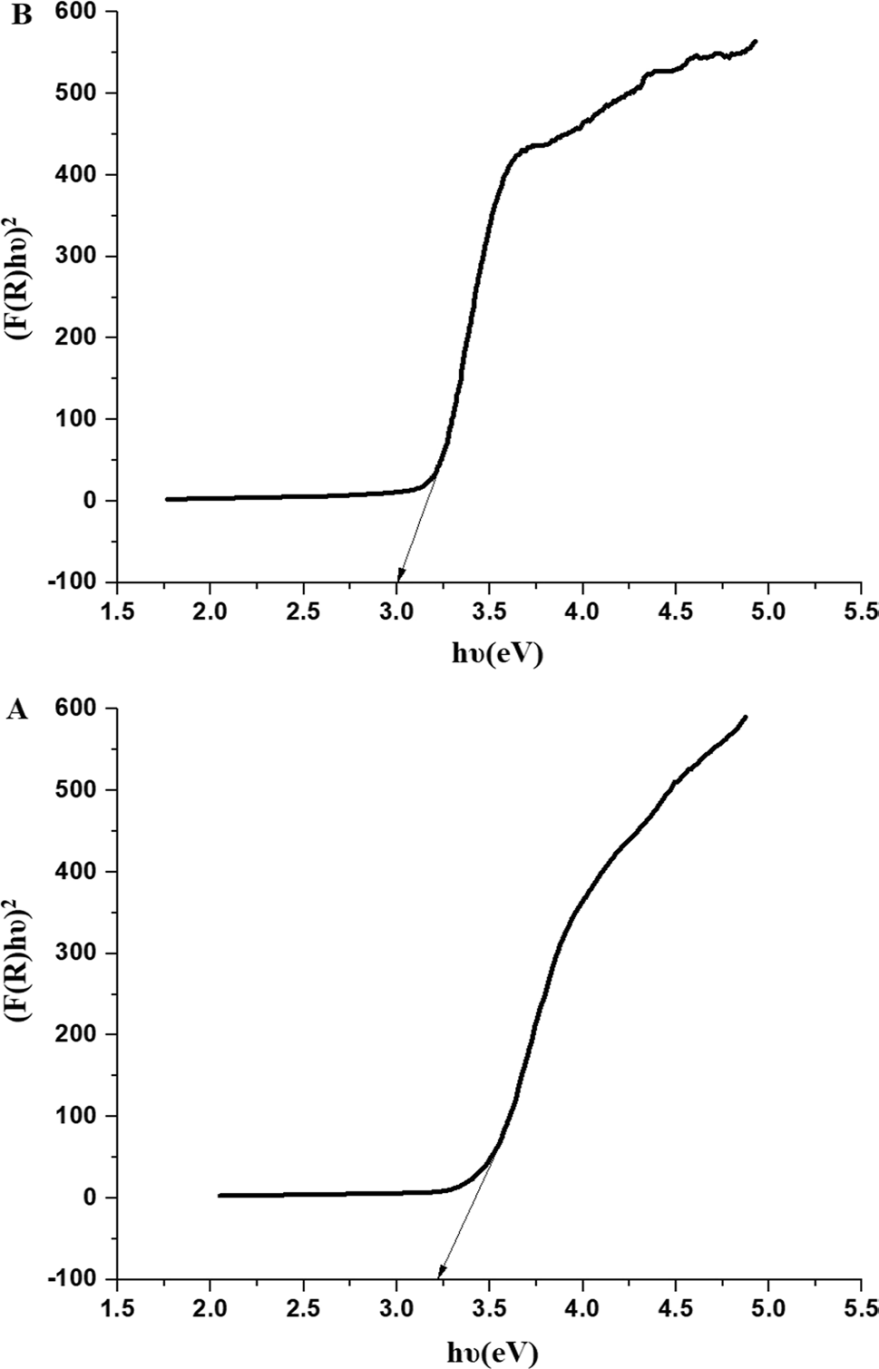
The optical energy gap of the EA600 (**A**) and EA800 (**B**) samples

A constant, Kubelka–Munk function, and an integer determined by the nature of the transition are represented by *K*_E_, *F*(*R*), and *N*, respectively. For direct allowed transitions, the value of *N* is 2, whereas for indirect allowed transitions, the value of *N* is 0.5. Figure 5A and B displays the plot of (*F*(*R*)*hυ*)^2^ versus hυ for the EA600 and EA800 samples, respectively. Accordingly, direct allowed transitions were found to be the most common in both the EA600 and EA800 samples. The calculation of the optical energy gap (*E*_g_) involves extrapolating each graph until (*F*(*R*)*hυ*)^2^ reaches 0. Consequently, the EA600 and EA800 samples exhibit optical energy gaps of 3.22 and 3.03 eV, respectively. Hence, the results confirmed that the smaller particle size leads to increased band gap energy. Smaller particle sizes can lead to an apparent increase in the band gap energy due to the confinement of charge carriers within the nanoscale dimensions [61].

**Fig. 6:**
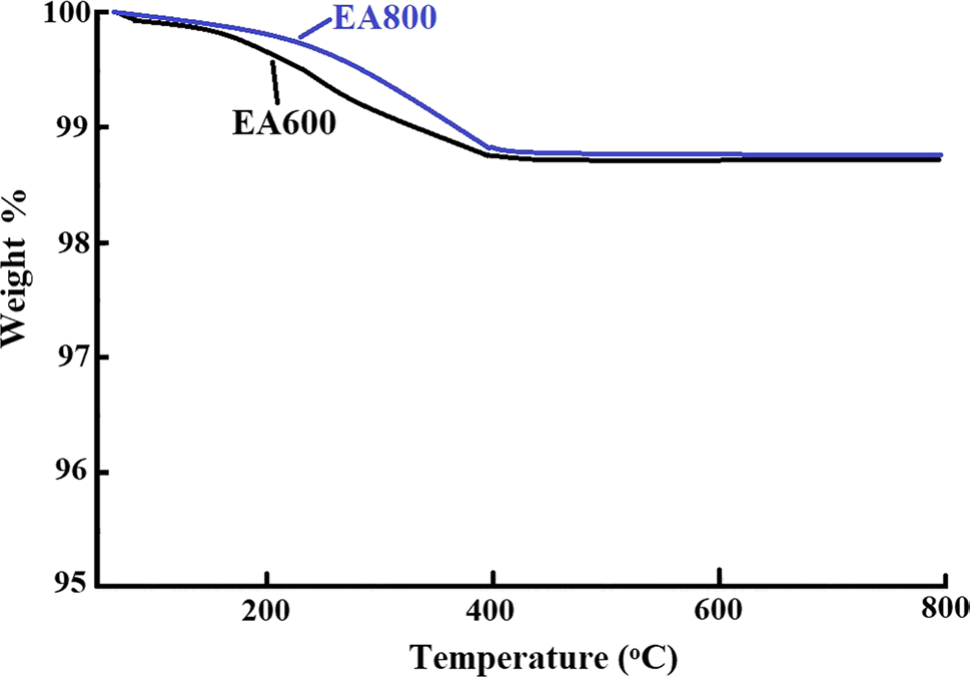
The thermogravimetric analysis of EA600 and EA800 samples

Barium titanate nanoparticles exhibit good thermal stability because they can withstand high temperatures without undergoing significant structural or chemical changes, as shown in Fig. 6. The thermogravimetric step, which is located in the range from 25 to 400 °C, can be attributed to the loss of adsorbed water molecules with a weight loss percentage of 1.35 and 1.14% in the cases of EA600 and EA800, respectively.

### Photocatalytic decomposition of malachite green dye using the synthesized barium titanate nanoparticles

#### Effect of malachite green dye solution pH

**Fig. 7:**
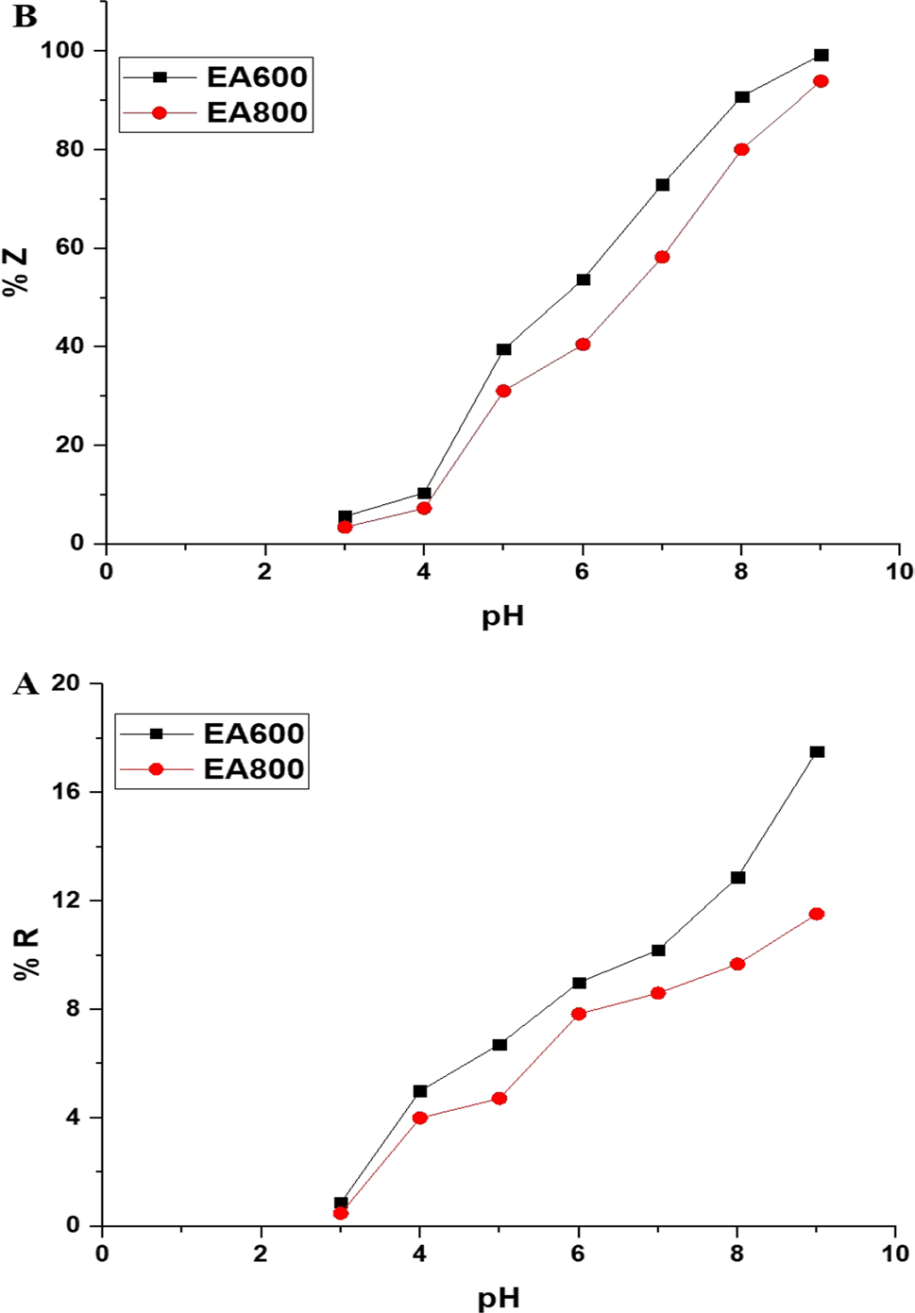
The influence of solution pH on the percentage of adsorption in a dark environment (**A**) and photocatalytic decomposition (**B**) of malachite green dye using the EA600 and EA800 samples

Figure 7A illustrates how pH impacts the percentage of adsorption (% *R*) of malachite green dye in a dark environment using the EA600 and EA800 samples. The percentage of adsorption (% *R*) of malachite green dye in a dark environment using the EA600 and EA800 samples is estimated by Eq. (3) [62].

3 $$\% R = \frac{{C_{{\text{o}}} - C_{{\text{d}}} }}{{C_{{\text{o}}} }} \times 100$$

where *C*_d_ (mg/L) represents the resultant concentration of malachite green dye after being adsorbed in a dark environment. *C*_o_ (mg/L) denotes the initial concentration of malachite green dye. Furthermore, the percentage of adsorption of malachite green dye in a dark environment using the EA600 and EA800 samples is 17.52 and 11.52%, respectively. These adsorption percentages are very weak, which confirms later that what will happen to the malachite green dye in the event of exposure to ultraviolet light is photocatalytic decomposition only. Figure 7B illustrates how pH impacts the percentage of photocatalytic activity of the EA600 and EA800 samples towards malachite green dye in the presence of ultraviolet light. The percentage of the photocatalytic decomposition of malachite green dye employing the EA600 and EA800 samples is calculated using Eq. (1), as previously mentioned. The decomposition percentage that is calculated using Eq. (1) does not depend on adsorption. Hence, this confirms that the observed decrease in the concentration of malachite green dye under the effect of ultraviolet light is due to the photocatalytic decomposition only. For the EA600 sample, a gradual increase in pH from 3 to 9 resulted in a progressive rise in the percentage of photocatalytic activity from 5.65 to 99.27%. Similarly, for the EA800 sample, a gradual increase in pH from 3 to 9 resulted in a progressive rise in the percentage of photocatalytic activity from 3.46 to 93.94%. The acidic medium surrounds the EA600 and EA800 samples with positive hydrogen ions, causing the expulsion of the cationic malachite green dye and subsequently decreasing the percentage of the photocatalytic activity [45, 46, 63]. Conversely, the basic medium envelops the EA600 and EA800 samples with negative hydroxide ions, causing the attraction of the cationic malachite green dye and subsequently increasing the percentage of the photocatalytic activity [45, 46, 63]. Based on these observations, the best pH value for achieving the maximum percentage of photocatalytic activity of the EA600 and EA800 samples towards malachite green dye is determined to be 9.

The percentages of photocatalytic activity of the EA600 and EA800 samples towards malachite green dye were compared with those of other photocatalysts such as CuO/TiO_2_, NiO/TiO_2_, CuCo_2_O_4_, chitosan/Ce/ZnO, EDTA/ZnO, Fe(III)-cross-linked alginate–carboxymethyl cellulose, cobalt oxide/citric acid, Dy_2_O_3_/SiO_2_, and lanthanide cerate, as given in Table 1 [64–71]. Consequently, we can assume that the EA600 and EA800 photocatalysts were highly efficient for the decomposition of the malachite green dye.

**Table 1:** Photocatalytic decomposition of the malachite green dye applying several photocatalysts

Photocatalyst	% *Z*	Amount of catalyst (g)	Concentration of dye (mg/L)	Volume of dye (mL)	Refs.
CuO/TiO_2_	96.00	0.1	20	100	[64]
NiO/TiO_2_	80.00	0.1	20	100	[64]
CuCo_2_O_4_	96.00	0.1	20	100	[65]
Chitosan/Ce/ZnO	87.00	0.03	5	25	[66]
EDTA/ZnO	94.10	0.002	3.6	100	[67]
Fe(III)-cross-linked alginate-carboxymethyl cellulose	98.80	0.1	10	50	[68]
Cobalt oxide/citric acid	91.20	0.05	10	100	[69]
Dy_2_O_3_/SiO_2_	71.08	0.03	5	50	[70]
Lanthanide cerate	70.50	0.048	24	50	[71]
EA600	99.27	0.05	25	100	This study
EA800	93.94	0.05	25	100	This study

#### Effect of UV irradiation time

**Fig. 8:**
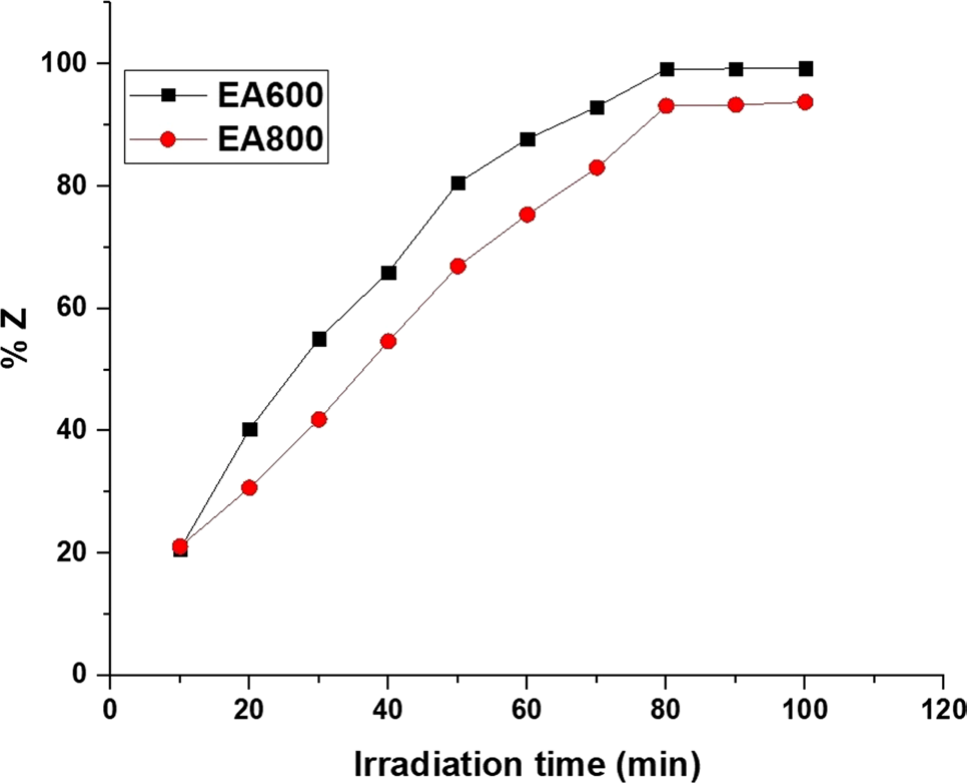
The influence of irradiation time on the percentage of photocatalytic activity of the EA600 and EA800 samples towards malachite green dye in the presence of UV light

Figure 8 illustrates how irradiation time impacts the percentage of photocatalytic activity of the EA600 and EA800 samples towards malachite green dye in the presence of UV light. For the EA600 sample, a gradual increase in the UV irradiation time from 10 to 80 min resulted in a progressive rise in the percentage of photocatalytic activity from 20.71 to 99.18%. Similarly, for the EA800 sample, a gradual increase in the irradiation time from 10 to 80 min resulted in a progressive rise in the percentage of photocatalytic activity from 21.11 to 93.13%. Subsequently, as the applied irradiation time was extended from 80 to 100 min, the percentage of photocatalytic activity exhibited by the EA600 and EA800 samples towards malachite green dye remained relatively stable due to the saturation of active sites. Based on these observations, the optimal irradiation time value for achieving the highest percentage of photocatalytic activity of the EA600 and EA800 samples towards malachite green dye is determined to be 80 min.

In the context of studying photocatalytic decomposition, the zero-order equation is not commonly used. The zero-order kinetics typically describe a reaction where the rate of the reaction is independent of the concentration of the reactants. However, in photocatalytic decomposition, the reaction rate is typically dependent on the concentration of the reactants. Consequently, the photocatalytic activity of both the EA600 and EA800 samples towards malachite green dye was studied applying the first-order and second-order kinetic models as described by Eqs. (4) and (5), respectively [69].

4 $$\ln \frac{{C_{{\text{d}}} }}{{C_{{\text{e}}} }} = K_{D1} t$$

5 $$\left( {\frac{1}{{C_{{\text{e}}} }} - \frac{1}{{C_{{\text{d}}} }}} \right) = K_{D2} t$$

**Fig. 9:**
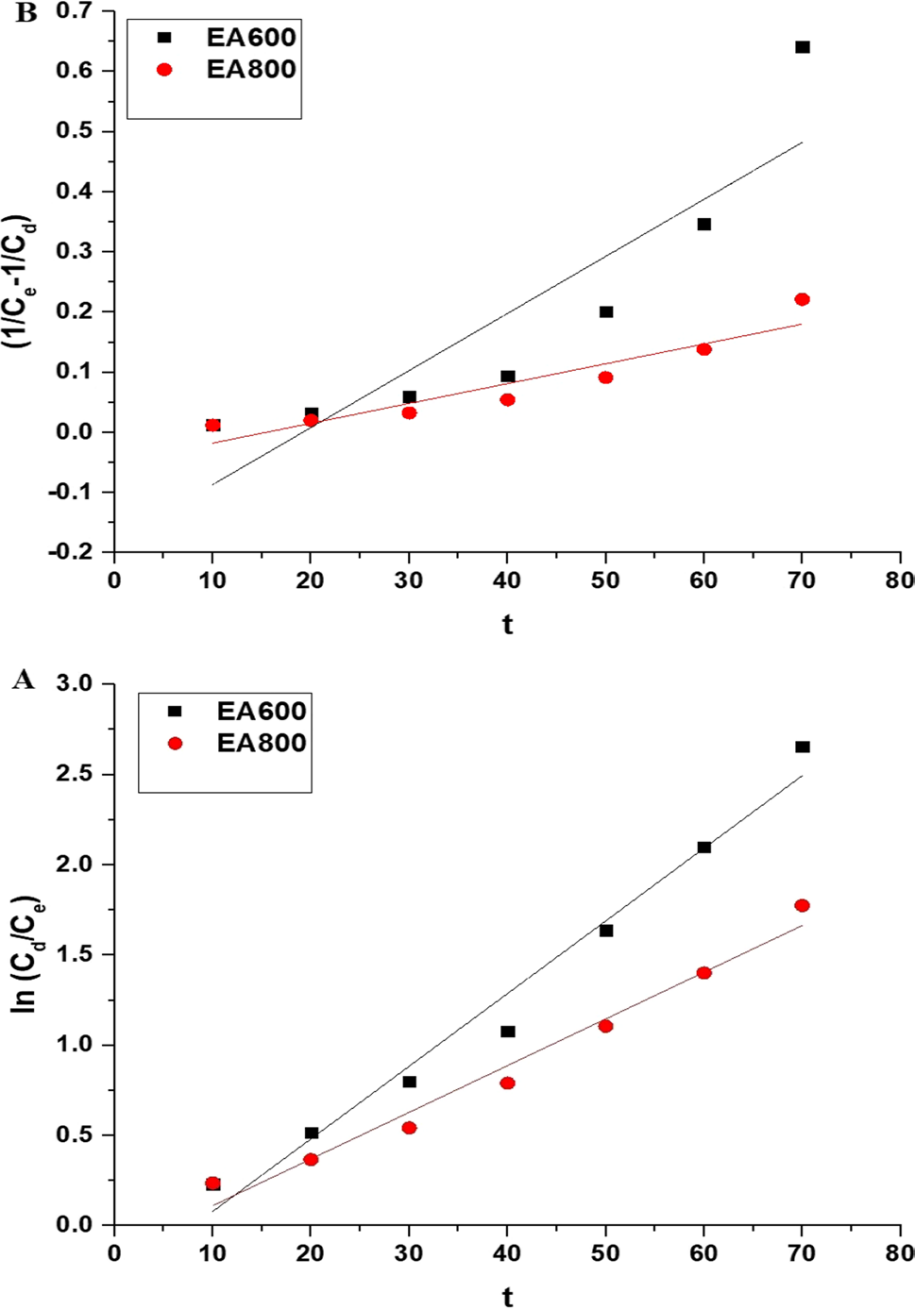
The first-order (**A**) and second-order (**B**) kinetic models for the photocatalytic activity of the EA600 and EA800 samples towards malachite green dye

The rate constant of the first order (*K*_*D*1_) is expressed in units of 1/min. Also, the rate constant of the second order (*K*_*D*2_) is expressed in units of L/mol min. Figure 9A and B illustrates the first-order and second-order kinetic models, respectively. Table 2 displays the calculated constants of the first- and second-order models. Since the correlation coefficients in the first-order case are higher than their counterparts in the second-order case, the photocatalytic activity of both the EA600 and EA800 samples towards malachite green dye adheres closely to a first-order kinetic model.

**Table 2:** The calculated constants of the first- and second-order kinetic models

Sample	First order		Second order	
	*K*_*D*1_ (1/min)	*R*^2^	*K*_*D*2_ (L/mol min)	*R*^2^
EA600	0.0403	0.9732	0.0095	0.7784
EA800	0.0259	0.9709	0.0033	0.8553

#### Effect of the amount of catalyst

**Fig. 10:**
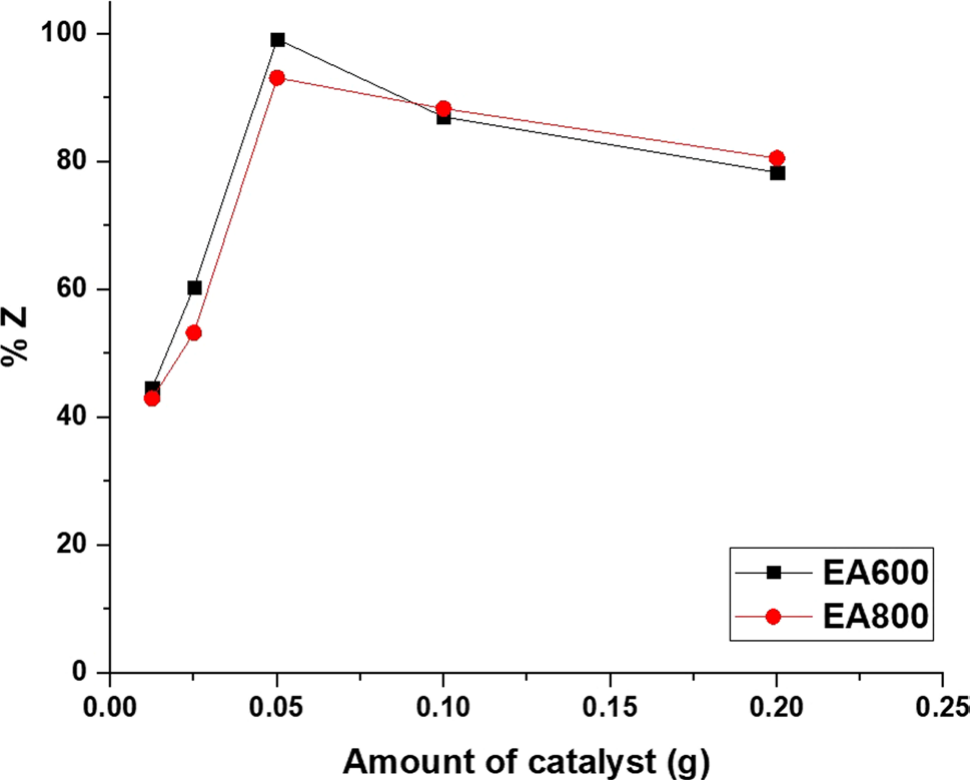
The influence of the amount of catalyst on the percentage of photocatalytic activity of the EA600 and EA800 samples towards malachite green dye in the presence of UV light

Figure 10 illustrates how the amount of catalyst impacts the percentage of photocatalytic activity of the EA600 and EA800 samples towards malachite green dye in the presence of ultraviolet light. For the EA600 sample, a gradual increase in the amount of catalyst from 0.0125 to 0.05 g resulted in a progressive rise in the percentage of photocatalytic activity from 44.53 to 99.18% as a result of the augmentation in free radicals [45, 46, 63]. Furthermore, when the amount of the BaTiO_3_ photocatalyst is extended from 0.05 to 0.20 g, the percentage of photocatalytic activity of the EA600 sample towards malachite green dye decreases from 99.18 to 78.32% owing to the resultant turbidity that obstructs the penetration of ultraviolet light into the solution [45, 46, 63]. For the EA800 sample, a gradual increase in the amount of catalyst from 0.0125 to 0.05 g resulted in a progressive rise in the percentage of photocatalytic activity from 42.93 to 93.13% as a result of the augmentation in free radicals [45, 46, 63]. Moreover, when the amount of photocatalyst is extended from 0.05 to 0.20 g, the percentage of photocatalytic activity of the EA800 sample towards malachite green dye reduces from 93.13 to 80.56% owing to the resultant turbidity that obstructs the penetration of ultraviolet light into the solution [45, 46, 63]. Based on these observations, the best amount of the catalyst for achieving the maximum percentage of photocatalytic activity of the EA600 and EA800 samples towards malachite green dye is determined to be 0.05 g. When the amount of photocatalyst is increased from 0.05 to 0.20 g, it might seem intuitive to expect an increase in the percentage of photocatalytic degradation of dye. However, in some cases, increasing the amount of photocatalyst beyond a certain point (in this case 0.05 g) can actually lead to a decrease in the efficiency of the photocatalytic process. This phenomenon can be explained by several factors:

1. Aggregation or clustering: When a higher amount of photocatalyst is used, the particles may have a tendency to aggregate or cluster together. This can limit the availability of active surface sites for dye adsorption and reaction. As a result, the effective surface area for photocatalytic degradation decreases, leading to a decrease in the overall efficiency.
2. Light absorption and scattering: Increased amounts of photocatalyst particles can result in a higher density of particles in the reaction mixture. This higher density can lead to increased light scattering and absorption within the solution, reducing the penetration of light to the photocatalyst surface. As a consequence, fewer photons reach the catalyst surface, resulting in reduced photocatalytic activity and lower degradation efficiency.
3. Catalyst Overloading: Increasing the amount of photocatalyst beyond a certain point can lead to catalyst overloading. This means that the available active sites on the catalyst surface become saturated, and adding more catalyst does not contribute to additional active sites for reaction. As a result, the overall photocatalytic degradation efficiency may plateau or even decrease as the excess catalyst does not contribute to the reaction [45, 46, 63].

#### Effect of primary concentration of malachite green dye

**Fig. 11:**
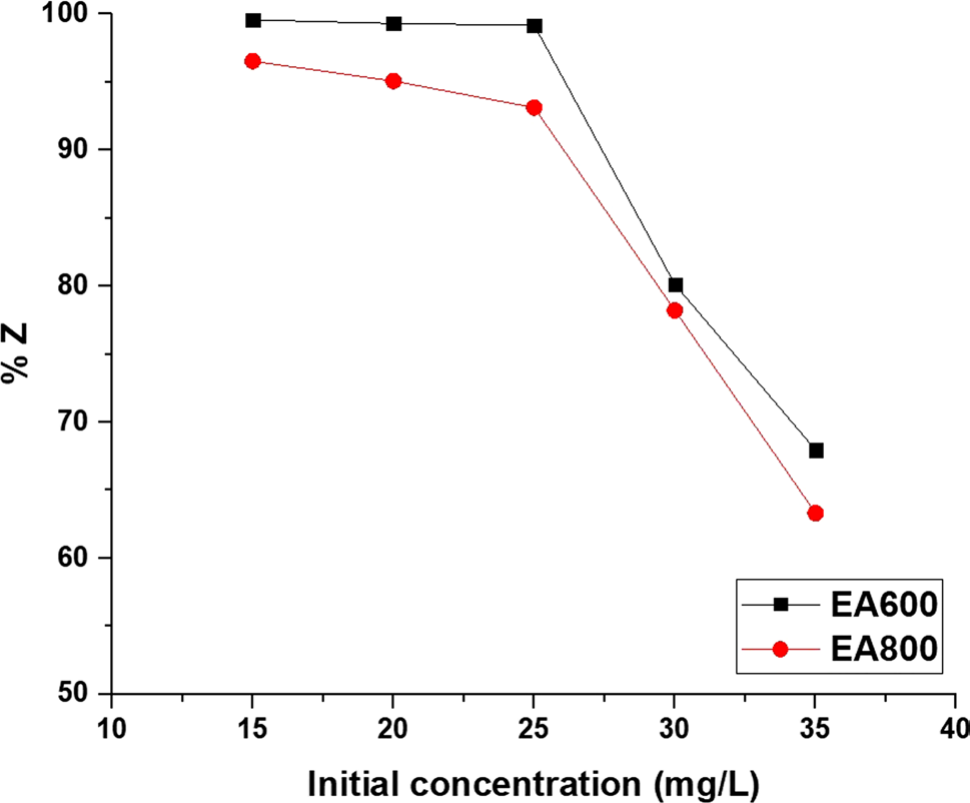
The influence of primary concentration of malachite green dye on the percentage of photocatalytic activity of the EA600 and EA800 samples in the presence of ultraviolet light

Figure 11 illustrates how the primary concentration of malachite green dye impacts the percentage of the photocatalytic activity of the EA600 and EA800 samples in the presence of ultraviolet light. For the EA600 sample, a gradual increase in the primary concentration of malachite green dye from 15 to 35 mg/L resulted in a progressive decrease in the percentage of photocatalytic activity from 99.58 to 67.96%. For the EA800 sample, a gradual increase in the primary concentration of malachite green dye from 15 to 35 mg/L resulted in a progressive decrease in the percentage of photocatalytic activity from 96.53 to 63.32%. There are three reasons behind the decline in catalytic activity when the malachite green dye concentration increases [72, 73]:

1. The reduction in the path length of generated photons occurs as the concentration of the malachite green dye increases.
2. At higher malachite green dye concentrations, malachite green dye absorbs photons more prominently than the catalyst.
3. The high concentration of the malachite green dye led to a decrease in the ratio of hydroxyl free radicals to malachite green dye molecules.

#### Effect of regeneration, reusability, and stability of catalysts

**Fig. 12:**
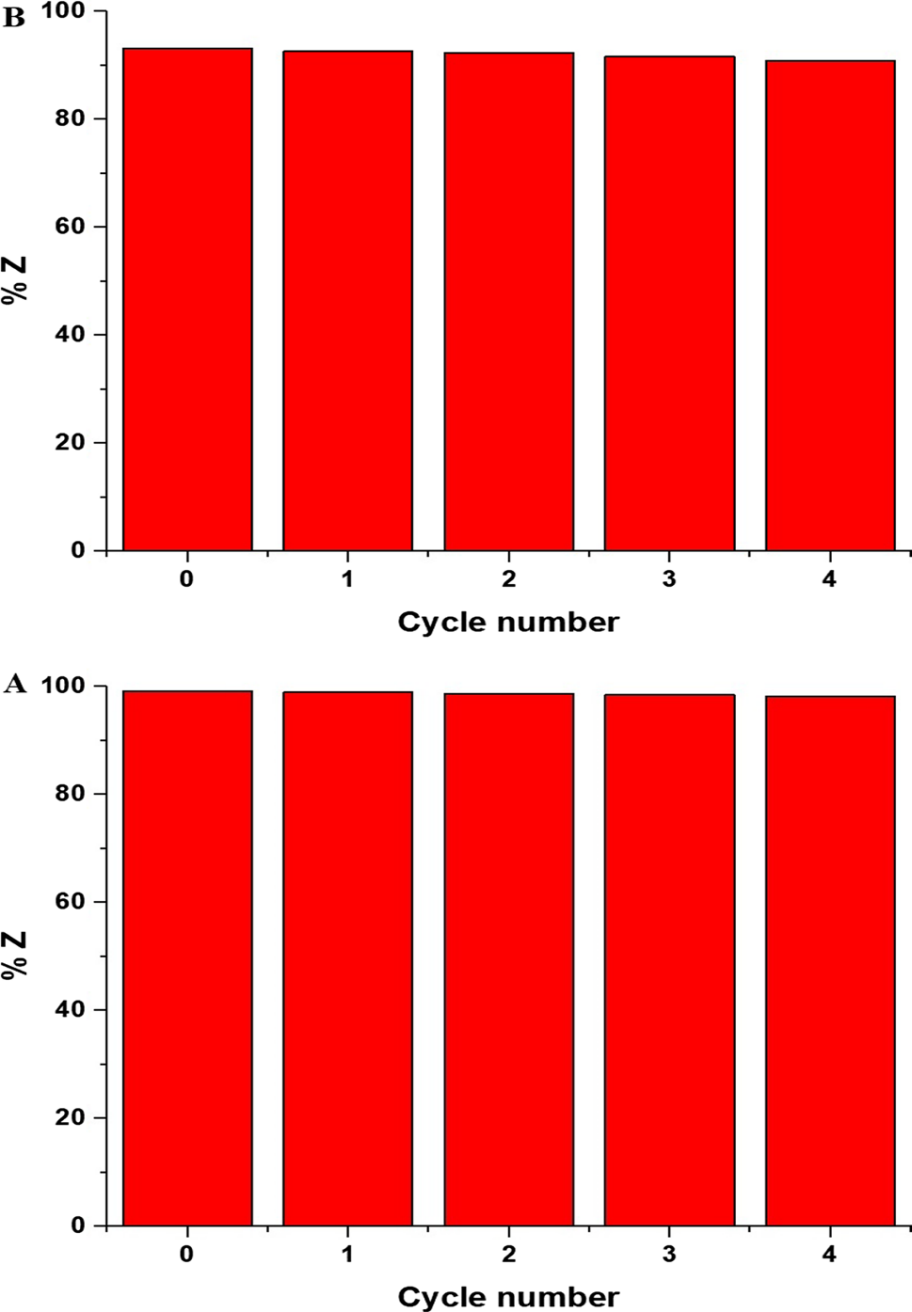
The influence of reusability of the EA600 (**A**) and EA800 (**B**) catalysts on the percentage of the photocatalytic decomposition of malachite green dye

The BaTiO_3_ nanoparticles obtained from the initial photocatalytic decomposition experiment (first cycle) were employed in subsequent photocatalytic decomposition experiments. In this investigation, the separated BaTiO_3_ nanoparticles were rinsed with ethanol and distilled water several times, followed by drying at 65 °C for 2 h utilizing a vacuum oven. Furthermore, the dried BaTiO_3_ nanoparticles were then reused in a second photocatalytic decomposition experiment using identical experimental parameters as those employed in the first cycle. Additionally, the reusability of the BaTiO_3_ nanoparticles was evaluated for four sequential cycles. Besides, Fig. 12A and B depicts the plot illustrating the percentage of malachite green dye degradation versus the cycle number, using the EA600 and EA800 products, respectively. Additionally, the obtained results demonstrated a marginal variation in the decomposition percentage of malachite green dye after four cycles, affirming the efficacy of the fabricated BaTiO_3_ nanoparticles and their potential for repeated utilization with nearly identical efficiency in decomposing the malachite green dye. To study the stability of the EA600 and EA800 catalysts, an XRD analysis was performed before and after photocatalytic decomposition (figures omitted for brevity). The results showed that there was no difference in the positions and intensity of the peaks, which confirms the stability of the used catalysts. In addition, the inductively coupled plasma (ICP) confirmed that there is no ion leaching from the barium titanate in the filtrate during the degradation processes.

#### Mechanism of photocatalytic decomposition of malachite green dye

**Scheme 2.**
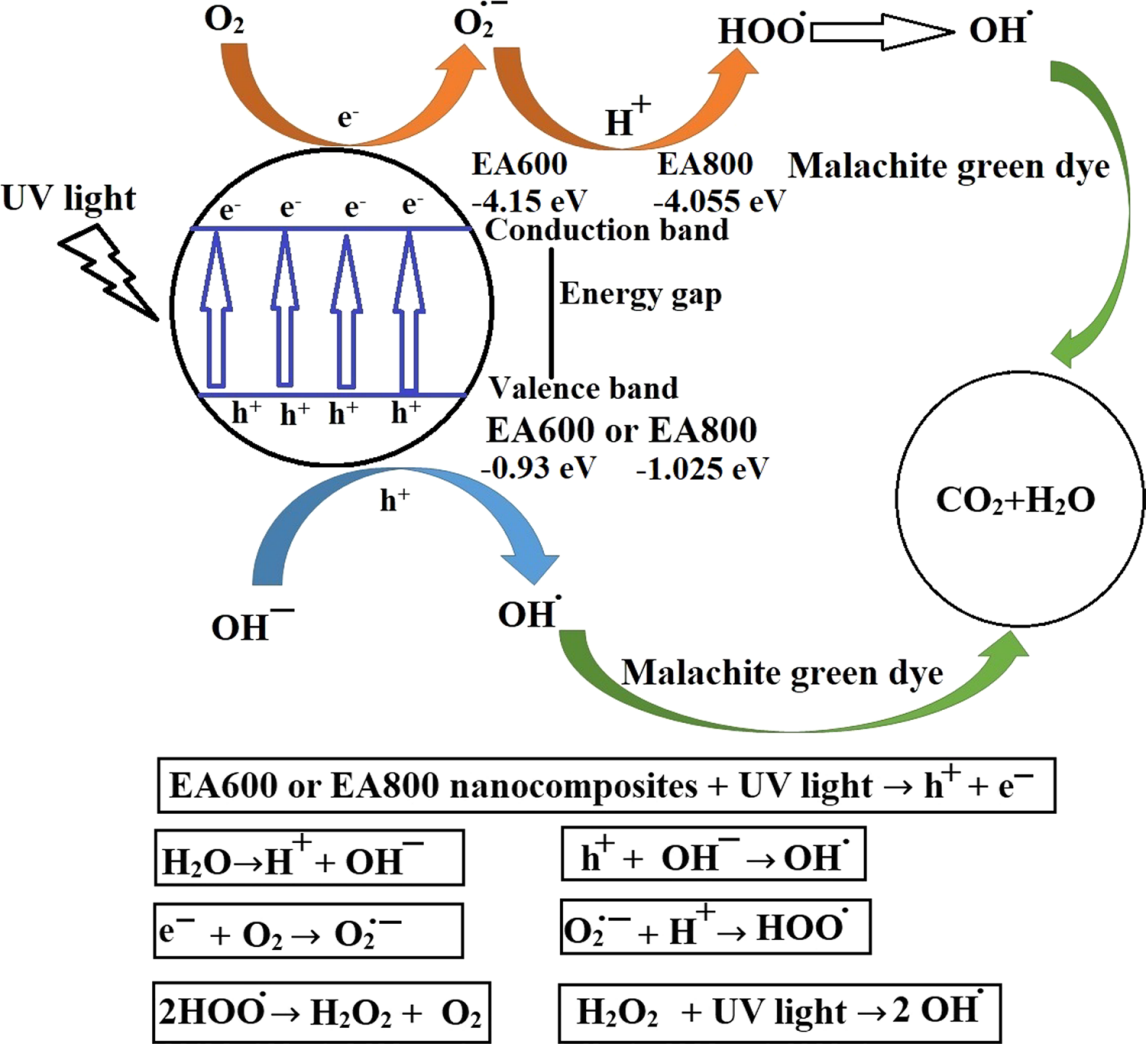
The mechanism of photocatalytic decomposition of malachite green dye by the EA600 and EA800 nanocomposites

Scheme 2 illustrates the process by which the EA600 and EA800 nanocomposites facilitate the photocatalytic decomposition of malachite green dye. If the barium titanate nanoparticles are exposed to ultraviolet radiation, the EA600 or EA800 nanoparticles undergo a transition wherein certain electrons move from the valence band towards the conduction band. Consequently, in the conduction band, electrons are generated, while in the valence band, holes are formed. The formed holes then interact with negatively charged hydroxide ions, resulting in the creation of hydroxyl radicals (OH^.^). Simultaneously, the generated electrons combine with the oxygen molecules to produce oxygen anion radicals (O_2_^·**−**^). Furthermore, the oxygen anion radicals combine with positive hydrogen ions to give rise to peroxide radicals (HOO^·^), which, in the presence of UV light, transform into hydroxyl radicals. Ultimately, these hydroxyl radicals actively degrade the malachite green dye, converting it into harmless substances like carbon dioxide and water [45, 46, 63]. To verify the process of photocatalytic decomposition of the malachite green dye through the evolution of carbon dioxide gas, a solution of barium chloride was added, and turbidity was observed. In addition, the clear change in the color of the malachite green dye over time also confirms the continuation of photocatalytic decomposition of the malachite green dye applying the synthesized catalysts.

**Fig. 13:**
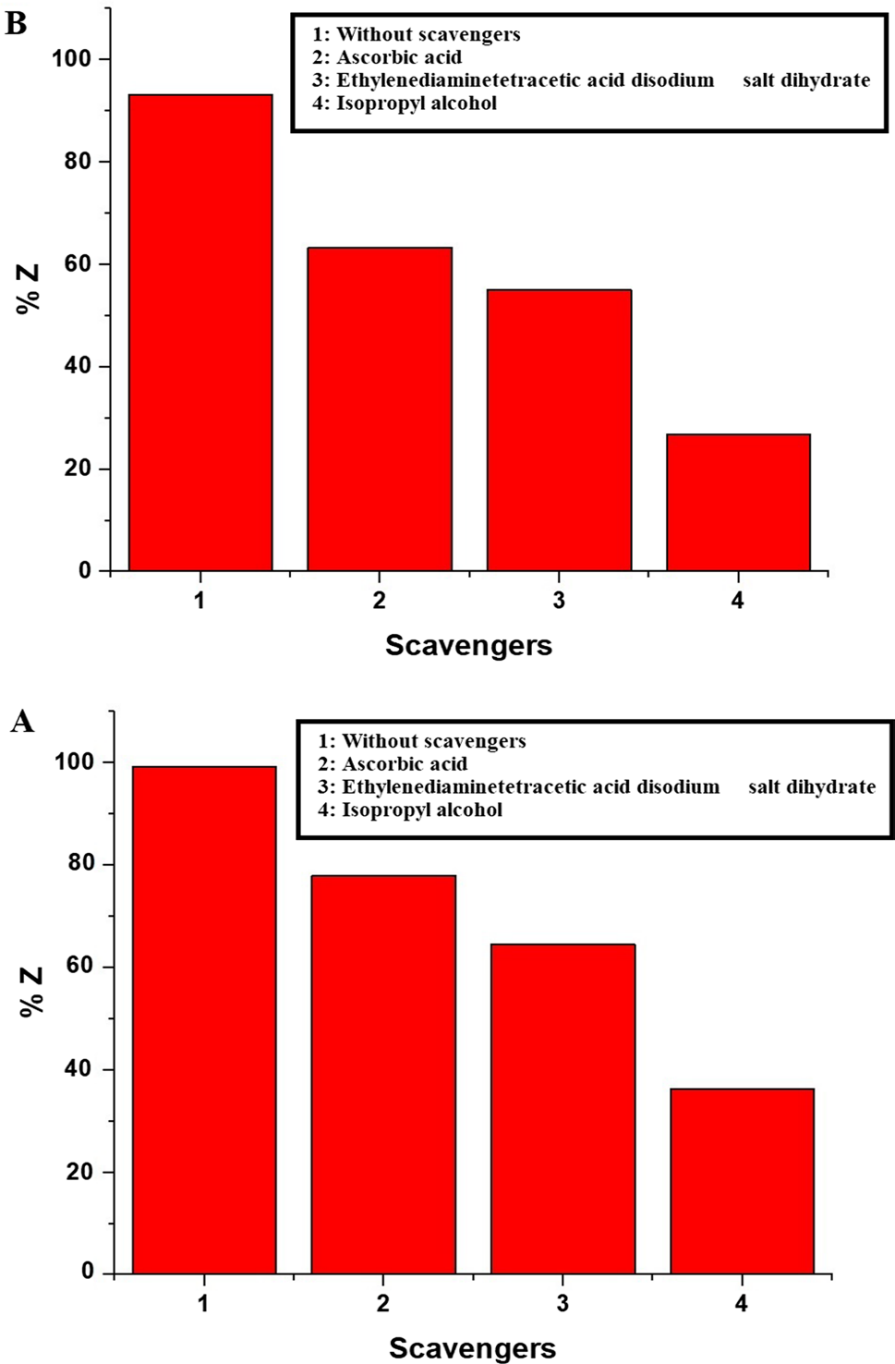
The effect of scavengers on the photocatalytic activity of the EA600 (**A**) and EA800 (**B**) samples towards malachite green dye

To validate the aforementioned mechanism, ascorbic acid served as an electron and oxygen anion free radical scavenger. Additionally, isopropyl alcohol and ethylenediaminetetraacetic acid disodium salt dihydrate were employed as scavengers for hydroxyl radicals and holes, respectively. In addition, Fig. 13A and B displays the effect of scavengers on the photocatalytic activity of the EA600 and EA800 samples towards malachite green dye, respectively. The obtained outcomes demonstrated a reduction in the decomposition percentage, providing evidence for the involvement of oxygen anion radicals, hydroxyl radicals, holes, and electrons in the decomposition process of malachite green dye, as depicted in Scheme 2 [18].

## Conclusions

Barium titanate (BaTiO_3_) nanoparticles were facilely synthesized, by the Pechini sol–gel method at 600 and 800 °C, as an efficient photocatalysts for the removal of malachite green dye from aqueous media. The samples, which were synthesized at 600 and 800 °C, were abbreviated as EA600 and EA800, respectively. Additionally, the mean crystallite size of the EA600 and EA800 products is 14.83 and 22.27 nm, respectively. Furthermore, the best pH, irradiation time, and the quantity of nanoparticles for achieving the maximum percentage of photocatalytic activity of the EA600 and EA800 samples towards malachite green dye are determined to be 9, 80 min, and 0.05 g, respectively. The maximum percentages of photocatalytic activity of the EA600 and EA800 samples towards 100 mL of 25 mg/L of malachite green dye are 99.27 and 93.94%, respectively. The influence of regeneration and reusability demonstrated a marginal variation in the degradation percentage of malachite green dye after four consecutive cycles, affirming the efficacy of the fabricated BaTiO_3_ nanoparticles and their potential for repeated utilization with nearly identical efficiency in decomposing the malachite green dye.

## Data Availability

All the data generated or analyzed during this study are incorporated in this article.
